# Identifying cognitive‐affective mechanisms underlying disability in episodic migraine: Using the fear avoidance model to examine interactions

**DOI:** 10.1111/head.14988

**Published:** 2025-06-17

**Authors:** Janosch Fox, Charly Gaul, Merle Kuhlencord, Nicolina Peperkorn, Julia Ohse, Joshua Krutzki, Youssef Shiban

**Affiliations:** ^1^ Department of Psychology PFH Göttingen Göttingen Germany; ^2^ Department of Psychiatry and Psychotherapy University Medical Center Göttingen Göttingen Germany; ^3^ Medical Faculty University of Duisburg‐Essen Essen Germany; ^4^ Headache Center Frankfurt Frankfurt Germany

**Keywords:** depression, episodic migraine, fear avoidance model, fear of attacks, interaction, mediation analyses

## Abstract

**Objective:**

Using the fear‐avoidance model (FAM) as a theoretical framework, this study examined the interactions between empirical factors contributing to disability in episodic migraine. It was tested whether pain catastrophizing, fear of attacks, and depressiveness mediate the relationship between pain experience and disability.

**Background:**

Migraine is a prevalent primary headache disorder associated with significant impairment in daily life. Biological and psychosocial factors contribute to its impact; however, a comprehensive model explaining the mechanisms underlying migraine‐related disability is still lacking.

**Methods:**

A cross‐sectional online survey was conducted between October 2023 and March 2024 to collect sociodemographic and clinical characteristics of patients with episodic migraine. To evaluate the proposed links within the FAM, two confirmatory path analyses were performed. In Model 1, the Pain Disability Index was used to quantitatively measure subjective aspects of disability. In Model 2, the Migraine Disability Assessment questionnaire was used to assess quantitative aspects of disability. Mediators derived from the FAM included: pain experience (attack frequency and pain intensity), dysfunctional cognitive pain processing (pain catastrophizing), and dysfunctional cognitive‐affective response to pain (fear of attacks and depressiveness).

**Results:**

Both path analyses demonstrated good model fit. The explained variance of migraine‐related disability was 28% (adjusted *R* ^*2*^ = 0.28) in both models, indicating large effect sizes. Attack frequency (standardized path coefficient [β] = 0.21, *p* < 0.001; β = 0.45, *p* < 0.001), pain intensity (β = 0.27, *p* < 0.001; β = 0.16, *p* < 0.001), fear of attacks (β = 0.12, *p* = 0.006; β = 0.13, *p* = 0.004), and depressiveness (β = 0.34, *p* < 0.001; β = 0.12, *p* = 0.006), were identified as independent predictors of disability in both models (Model 1; Model 2). Consistent with the hypothesis, an indirect pathway from attack frequency and pain intensity to disability via pain catastrophizing, fear of attacks, and depressiveness was observed in both models.

**Conclusion:**

This study emphasizes the important role of (potentially modifiable) dysfunctional cognitive pain processing and provides empirical evidence for the theoretical assumptions of the FAM. Attack frequency, pain intensity, fear of attacks, and depressiveness were found to be independent predictors of subjectively and quantitatively measured disability in episodic migraine. Pain catastrophizing was identified to be a crucial cognitive‐affective factor mediating the relationship between pain experience and disability.

## Plain Language Summary

Migraine is a common condition that can affect daily life, even between attacks. However, it is unclear which mechanisms lead to this impairment, and a comprehensive explanatory model is lacking. This study found that pain catastrophizing, a way of thinking where people expect the worst and feel helpless about pain, as well as fear of attacks and depressiveness, may be important mediators between the pain experience and disability.

**Abbreviations**
AGFI: adjusted goodness‐of‐fit
CFI: Comparative Fit Index
CHS: Cluster Headache Scales
DASS: Depression, Anxiety and Stress Scales
*df*
: degrees of freedom
FAM: fear‐avoidance model
IHS: International Headache Society
IQR: interquartile range
MIDAS: Migraine Disability Assessment
NRS: numeric rating scale
PCS: Pain Catastrophizing Scale
PDI: Pain Disability Index
RCT: randomized controlled trial
RMSEA: root mean square error of approximation
SD: standard deviation
SRMR: standardized root mean square residual

## INTRODUCTION

Migraine is a highly prevalent primary headache disorder, affecting ~14% of the global population.^1^ Although acute and preventive treatments can help manage migraine attacks, the condition often persists for life and is currently incurable. Due to symptoms during attacks (ictal), as well as between attacks (interictal), migraine can have a profound impact on quality of life. Impairment significantly affects multiple domains of daily life, including occupational, academic, social, leisure, and family responsibilities. Migraine is the second leading cause of years lived with disability among all diseases,^1^ which underscores the far‐reaching impact not only on individuals, but also on public health systems. Furthermore, migraine is bidirectionally associated with psychiatric disorders,^2^, ^3^ in particular major depression and anxiety disorders, which can worsen the course of the disease, complicate its management, and increase the burden.^4^

### Disability is more than attack frequency

Key headache characteristics, such as attack frequency or accompanying symptoms (i.e., nausea, photophobia, and phonophobia), contribute to migraine‐related disability,^5^, ^6^, ^7^ while only partially explaining the functional impairment experienced by patients. For instance, a study analyzing data from three population‐based cohorts to evaluate the reliability and validity of the Migraine Disability Assessment (MIDAS) questionnaire—a widely used self‐administered tool for assessing migraine‐related disability—found that headache frequency alone contributed only ~6% to the explained variance in MIDAS scores.^8^ Including accompanying symptoms, pain intensity, and demographic factors increased the explained variance to 22%, which statistically is only considered a medium effect size.^9^ This shows that headache characteristics alone are not sufficient to explain disability, as it must be understood as a multifactorial construct.^10^, ^11^

In line with the biopsychosocial model of illness,^12^ extensive research has demonstrated that psychological factors can significantly modulate pain disorder outcomes.^13^ In the context of migraine, several psychological factors have been identified as important contributors to disability. These include psychological characteristics such as negative affect, anxiety, and depression,^2^, ^4^, ^14^, ^15^ maladaptive pain‐related cognitions like pain catastrophizing and fear of pain,^3^, ^16^, ^17^ as well as dysfunctional behaviors, including dysfunctional avoidance strategies.^18^ However, the mechanisms by which these psychological factors interact with each other and with headache characteristics are not yet fully understood.

### The fear‐avoidance model (FAM)

The FAM^19^, ^20^, ^21^ provides a theoretical framework based on a biopsychosocial understanding of illness that could substantially improve the understanding of the underlying mechanisms of disability. The FAM challenges previous paradigms that attributed disability solely to the severity of pain by emphasizing the disabling influence of cognitive, affective, and behavioral factors, which modulate the perception of pain.

According to the FAM, dysfunctional cognitive processing of actual or anticipated pain—particularly pain catastrophizing—can trigger dysfunctional cognitive‐affective responses, such as heightened fear of pain and depressiveness. These responses, in turn, promote maladaptive behaviors, including avoidance, pain hypervigilance, and social withdrawal. Over time, these patterns lead to functional impairment and contribute to long‐term disability.

### Research gap

To date, it is unclear which mechanisms underlie migraine‐related disability. Research has shown that attack frequency, pain intensity, pain catastrophizing, fear of attacks, and depressiveness are among the contributing factors; however, studies have predominantly focused on the investigation of factors as isolated predictors of disability, neglecting their interactions and integration into an explanatory model.

### Aim of this work

To address this gap, the present study applied the FAM to investigate the interaction of cognitive‐affective factors in episodic migraine. Using confirmatory path analysis, the hypothesis was tested that pain catastrophizing mediates the relationship between attack frequency or pain intensity and disability, with fear of attacks and depressiveness as endogenous variables. To capture disability as a multifaceted construct encompassing both subjective and quantitative dimensions, two different assessment tools were used.

## METHODS

A systematic cross‐sectional survey with a prospective design was conducted between October 2023 and March 2024. Patients (aged ≥18 years) with a migraine diagnosis were invited to participate in an anonymous online survey. Diagnoses were reassessed using the criteria of the International Classification of Headache Disorders, third edition.^22^ Inclusion required: (i) headache attacks lasting 4–72 h if untreated, (ii) at least two characteristics of the following—unilateral localization, pulsating quality, moderate or severe intensity, aggravation by or causing avoidance of routine physical activity—and at least one accompanying symptom (nausea and/or vomiting, photophobia, and phonophobia). Exclusion criteria were: (i) not meeting the International Classification of Headache Disorders, third edition criteria for migraine, (ii) chronic migraine, and (iii) incomplete data. The survey was conducted via the LimeSurvey platform.^23^

### Recruitment

Recruitment for the study was carried out online through the websites of the German Migraine Patients Organization (MigräneLiga e.V. Deutschland), the German Migraine and Headache Society (DMKG e.V. Deutschland), and the Headache Center Frankfurt. Additionally, participants were made aware of the study via Instagram and Facebook.

### Ethics

According to the responsible Ethics Committee of the State Medical Association of Hesse, no ethical approval was required for the present study, as studies using anonymous questionnaires for data collection are exempt from review. Participants were informed that participation was voluntary and could be terminated at any time without giving reasons. Written informed consent was obtained from all participants before completing the questionnaire. No financial compensation was paid for participation in the study.

### Power analysis

To determine the sample size, a simulation‐based power analysis was conducted, as path analysis involves complex dependencies. The analysis was performed using *R* (version 4.4.2; R Core Team, 2023), and the packages *semPower* (version 2.1.1)^24^ and *lavaan* (version 0.6–19).^25^ The adjusted goodness‐of‐fit (AGFI) was used as a criterion for determining the required sample size, as it is appropriate for models with limited degrees of freedom (*df*). It reflects the overall model fit rather than focusing on individual path coefficients. AGFI values >0.90 indicate an acceptable model fit.^26^ The power analysis was based on an AGFI target of 0.90, with an alpha level of 0.05, desired statistical power of 0.95, seven *df*, and six observed variables in the model. The analysis indicated that a sample size of 213 participants would be required to achieve the specified power.

### Measures

The survey included the German versions of the Depression, Anxiety, and Stress Scales (DASS) to assess psychological distress, the Pain Catastrophizing Scale (PCS) to assess dysfunctional pain processing, and the subscale “fear of attacks” of the Cluster Headache Scales (CHS) to assess fear of attacks. Migraine‐related disability was assessed with the Pain Disability Index (PDI) and the MIDAS questionnaire. Pain intensity was measured using a numeric rating scale (NRS). Additionally, headache‐related clinical characteristics (e.g., attack frequency and duration of illness) and sociodemographic data were recorded. Sex was assessed with the options: “male,” “female,” or “diverse.”

#### The PDI

The PDI^27^ measures functional impairment due to pain based on qualitative ratings provided by the patient. It consists of seven items that assess the degree to which pain interferes with functioning in seven key life domains: family/home responsibilities, recreation, social activity, occupation, sexual behavior, self‐care, and life‐support activity. Each item is scored on a scale of 0 (“no disability”) to 10 (“complete disability”), resulting in a total score between 0 and 70 that reflects overall disability. This score can be converted into a percentage (0–100%) using the following formula: (sum score of raw values/70) × 100. Higher scores represent greater disability. Although there is no definitive limit, scores ≥33 can be considered an indication of excessive pain‐related impairment.^28^, ^29^, ^30^, ^31^ Regarding psychometric properties, a study by Tait et al.^32^ supports the PDI's reliability as well as its concurrent and criterion‐related validity.

#### The MIDAS

The MIDAS^33^ is a brief questionnaire that assesses the impact of migraine on a patient's daily life over the past 3 months. It includes five items evaluating time lost due to headache attacks in three domains: work or school, household work, and social or leisure activities. The total score, derived by summing days missed in each domain, categorizes disability into four grades, from “minimal” (Grade I) to “severe” (Grade IV). The MIDAS was translated and adapted for its use in Germany by Benz et al.,^34^ who recommend its use in research due to the questionnaire's high reliability and validity.

#### The PCS

The PCS^16^ is a widely used tool that evaluates dysfunctional cognitive and affective responses to pain. It consists of 13 items, grouped into three subdomains: rumination, magnification, and helplessness. Patients rate each item on a 5‐point Likert scale ranging from 0 (“not at all”) to 4 (“all the time”), yielding a total score between 0 and 52. Higher scores indicate greater levels of pain catastrophizing, with sum scores ≥30 considered pathological, as this threshold represents the 75th percentile among patients with chronic pain.^35^ A meta‐analysis revealed excellent internal consistency and test–retest reliability for the PCS.^36^

#### The DASS

The German version of the DASS^37^, ^38^ consists of three self‐report scales designed to measure psychological distress. The subscales are depression, anxiety, and stress. Each consists of 14 items, scored on a 4‐point Likert scale from 0 (“do not apply to me at all”) to 3 (“applied to me very much”). Scores for each subscale are summed, with higher scores reflecting greater severity of symptoms. According to the authors, the exclusion of somatic items makes the questionnaire suitable for the assessment of psychological distress in the research and treatment of patients with pain.^39^ The DASS is a reliable tool with internal consistencies ranging from 0.78–0.82 (anxiety) and 0.81–0.89 (stress) to 0.91 (depression).

#### Fear of attacks

The assessment of fear of attacks was based on the “fear of attacks” subscale of the CHS,^40^ as no migraine‐specific questionnaire was available at the time of study planning.

The “fear of attacks” subscale comprises six items designated to evaluate individuals’ fear of experiencing a cluster headache attack. Responses on this scale are recorded using a 5‐point Likert scale to indicate their level of agreement for each of the items (1 = “strongly disagree,” 2 = “disagree,” 3 = “neither agree nor disagree,” 4 = “agree,” 5 = “strongly agree”). For the present study, only items that did not explicitly reference cluster headache symptoms and specifically addressed the fear of attacks were included. The items “I am worried that an attack might occur,” “I panic when I think about the next attack,” and “I am worried about the next attack,” were used to construct the scale. This approach was chosen to assess the frequency and dimensional structure of fear of attacks in the migraine population.

#### Pain intensity

The NRS (as described by Hartrick et al.^41^) is a self‐report pain screening tool that has been widely used in clinical settings to assess pain levels.^41^ When using this scale, patients are asked to rate the pain they experience on a scale ranging from 0 to 10, with 0 representing “no pain” and 10 representing “the worst pain imaginable.”^41^ Literature suggests that the NRS should be used in preference to other methods of measuring pain intensity.^42^, ^43^, ^44^

### Statistical Analysis

Statistical analyses were performed using the IBM Statistical Package for the Social Sciences (SPSS) and SPSS Amos (version 28; IBM Corp., Armonk, NY, USA). To ensure the integrity and reliability of the analysis, participants with incomplete data sets were excluded. To reduce the risk of measurement errors, we inspected all data points of the sociodemographic and clinical parameters for outliers. Although some data points were identified as outliers, the values were plausible, so no data points were excluded. Measures of central tendency (mean or median), measures of variability (standard deviation [SD] or interquartile range [IQR]), and frequency distributions (absolute [*N* or *n*] and relative frequency [%]) were used to summarize the characteristics of the study population and outcomes. The distribution of continuous and discrete data was assessed visually using histograms and Q–Q plots. Only attack frequency showed a left‐skewed, heavy‐tailed distribution (skewness = 1.67, standard error = 0.12; kurtosis = 5.00, standard error = 0.25).

Zero‐order correlations were computed to examine the bivariate relationships among the clinical characteristics and to gain a preliminary understanding of the data structure. The determination of correlation coefficients also facilitated the assessment of potential multicollinearity, which is a critical assumption for conducting path analyses. For the estimation of correlations between the interval‐scaled variables, Pearson's correlation coefficients were calculated; for ordinal‐scaled data, Spearman's correlation coefficients were used. The effect sizes are reported according to the conventions set by Cohen^9^: a correlation coefficient of ≥0.10 is considered a weak correlation, ≥0.30 a moderate correlation, and ≥0.50 a strong correlation; *R* ^*2*^ ≥ 0.02 is considered a small, *R* ^*2*^ ≥ 0.13 a moderate, and *R* ^*2*^ ≥ 0.26 a strong effect size.

**FIGURE 1:**
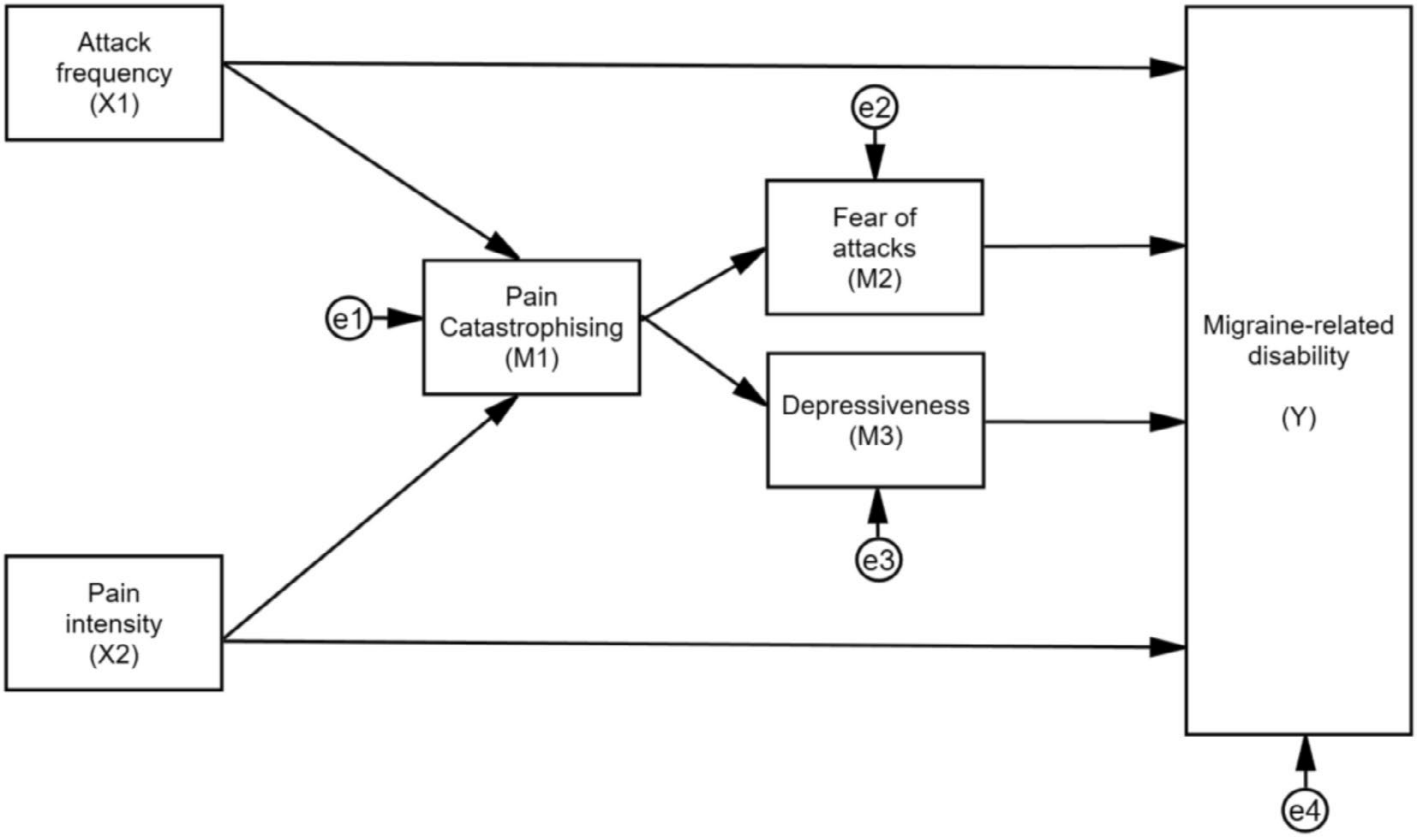
Conceptual framework of the mediation model. The rectangular boxes represent observed variables, arrows represent paths between variables, and the circular “e” symbols represent error terms.

A serial multiple mediator model^45^ (Figure 1) was used to test the hypothesis. The model was specified based on the FAM and examined the relationships between attack frequency (X1), pain intensity (X2), and disability (Y), with pain catastrophizing (M1), fear of attacks (M2), and depression (M3) as mediators. Error terms (e1–e4) were included to account for unexplained variance. The mediation model was then evaluated using two recursive path analyses, a regression‐based multivariate statistical approach, with the PDI (Model 1) or the MIDAS (Model 2) as the dependent variable. The analysis involved a stepwise procedure with multiple regressions: (i) a regression with X as the predictor and Y as the dependent variable, (ii) a regression with X as the predictor and M as the mediator, and (iii) a regression with X and M as the predictors and Y as the dependent variable. All variables in the models were observed. To ensure robust parameter estimates, bootstrapping was performed with 5000 samples and bias‐corrected 95% confidence intervals. Missing data were handled using listwise deletion. The proportion of explained variance (*R* ^*2*^) was calculated for the dependent variable, as well as for all endogenous variables. Standardized path coefficients (βs) were estimated for each path along with the corresponding significance levels. To determine the adequacy of the model fit, the results of the chi‐square test were examined alongside key fit indices, including the Comparative Fit Index (CFI; ≥0.90: acceptable; ≥0.95: good),^46^ root mean square error of approximation (RMSEA; ≤0.06: good),^46^ and standardized root mean square residual (SRMR; <0.08: acceptable; <0.06: good).^46^ Full‐information maximum likelihood was used to estimate parameters in the path analysis models.

We followed the methodology outlined by Fairchild et al.^47^ in calculating the proportion of the total explained variance in disability attributable to mediation. In this approach, the R^2^_mediation_ is calculated by determining the additional variance in the dependent variable (Y) explained by including the mediator (M). The mediation effect is calculated based on the difference in explained variance between different regression models: In Model 1, Y is predicted by both the independent variable (X) and the mediator (M); in Model 2, Y is predicted by M alone; in Model 3, Y is predicted by X alone. In our analysis, we included attack frequency (X1) and pain intensity (X2) as independent variables, disability (Y, measured using the PDI or MIDAS) as the dependent variable, and pain catastrophizing (M) as the mediator. The mediation effect size was then calculated by the change in the explained variance (*R* ^2^) across these models, using the following formula: *R* ^*2*^_mediation_ = *R* ^*2*^_Model2_ − (*R* ^*2*^_Model1_ − *R* ^*2*^_Model3_). A higher *R* ^*2*^_mediation_ indicates a stronger mediation effect.

All hypothesis tests were two‐tailed and considered significant at a *p* < 0.05.

## RESULTS

### Study population

**FIGURE 2:**
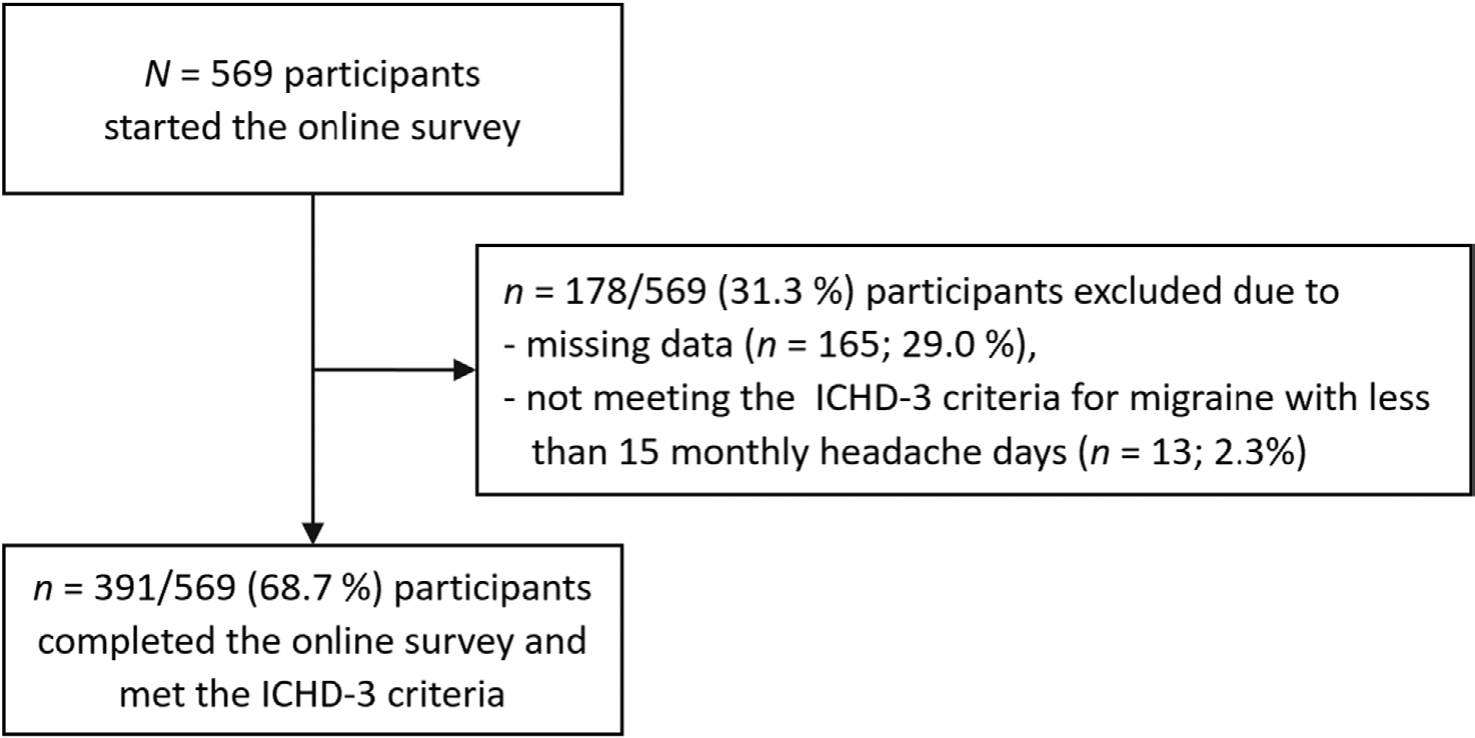
Flow chart of attrition. ICHD‐3, International Classification of Headache Disorders, third edition.

Out of the 569 initiated survey attempts, 178 (31.3%) cases were excluded due to incomplete data or failure to meet inclusion criteria, resulting in a final sample of 391 complete datasets used for the analysis (Figure 2). Data inspection did not reveal implausible values.

### Drop‐out analysis

A dropout analysis was conducted, including 177 (99.4%) of the 178 excluded datasets. The analysis revealed a slight but statistically significant difference in age between included datasets and dropouts (mean [SD] 41.4 [13.1] vs. 38.9 [12.8] years; *p* = 0.034; *d* = 0.19). Sex distribution was largely similar, with participants reporting female sex outnumbering those reporting male sex (94.6% vs. 5.4%). Two participants in the dropout group indicated diverse sex, while neither of the included data sets did. Regarding migraine frequency, no differences were found in the average number of attacks in the last 4 weeks (both groups median [IQR] 4.0 [2.0–8.0]; *p* = 0.596). As most dropouts exited the survey at an early stage, sum scores for the MIDAS, PDI, and PCS could only be calculated for <2%, which precludes a comparison of these values.

### Sociodemographic and migraine characteristics

The mean (SD, range) age of the participants was 41.4 (13.1, 18–76) years, with the majority identifying as female (94.6% [*n* = 370]). No participant indicated a sex other than male or female. Most of the participants (78.8% [*n* = 308]) had experienced migraine for >10 years. Aura symptoms were reported by 27.6%.

The median (IQR) number of headache days in the last 3 months was 15.0 (10.0–25.0) and the reported median (IQR) number of migraine attacks in the last 4 weeks was 4.0 (2.0–8.0).

Participants’ sociodemographic and migraine characteristics are summarized in Table 1.

**TABLE 1:** Sociodemographic and migraine characteristics of patients with episodic migraine (*N* = 391).

Characteristics	Value
Age, years, mean (SD, range)	41.4 (13.1, 18–76)
Sex, *n* (%)	
Female	370 (94.6)
Male	21 (5.4)
Education, *n* (%)	
Graduated 9th grade	8 (2.0)
Graduated 10th grade	31 (7.9)
High school diploma	53 (13.6)
Vocational training	132 (33.8)
Academic degree	167 (42.7)
Migraine history, *n* (%)	
<1 year	6 (1.5)
1–2 years	8 (2.0)
2–5 years	30 (7.7)
5–10 years	39 (10.0)
>10 years	308 (78.8)
Migraine with aura, *n* (%)	108 (27.6)
Attack frequency, median (IQR, range)	
Reported headache days in the last 3 months	15 (10.0–25.0, 1–42)
Reported migraine attacks in the last 4 weeks	4 (2.0–8.0, 0–28)

Abbreviations: IQR, interquartile range; *n*, number valid cases; SD, standard deviation.

### Migraine‐related disability and psychological distress

Measuring subjective aspects of disability, the PDI (converted to percentage) mean (SD) score was 44.9% (17.2%), with 73.9% of patients (*n* = 289) scoring ≥33, indicating significant disability. Measuring quantitative aspects of disability, the median (IQR) MIDAS score was 27.0 (14.0–50.0). MIDAS Grade I was observed in 7.7% of participants, Grade II in 9.2%, Grade III in 18.4%, and Grade IV in 64.7%.

The screening for *psychological distress* revealed that 22.0% of the patients (*n* = 86) experienced depressive symptoms (DASS depression score ≥10) and 30.2% (*n* = 118) experienced anxiety symptoms (DASS anxiety score ≥6). Pathological stress symptoms (DASS stress score ≥10) were reported by 34. 8% of patients (*n* = 136).

A *pain catastrophizing* score of >30 was observed in 21.2% of patients (*n* = 83), indicating that 78.8% of patients did not show pathological levels of catastrophizing according to the screening.

To assess the *fear of attacks* in the study population, we analyzed the items of the adapted CHS subscale “fear of attacks.” The answers were converted into a dichotomous format, where “strongly agree” and “agree” were combined into a single category labeled “agree,” while “strongly disagree,” “disagree,” and “neither agree nor disagree” were categorized as “disagree.” In all, 70.1% of the participants reported being afraid of an attack, 57.8% reported experiencing worry, and 30.7% reported panic when thinking about a future attack.

The clinical characteristics of the participants are summarized in Table 2.

**TABLE 2:** Clinical characteristics of patients with episodic migraine (*N* = 391).

Characteristics	Value	Value range
Pain intensity NRS score, mean (SD, range)	6.4 (1.6, 2–10)	0–10
PDI score, %, mean (SD, range)	44.9 (17.2, 0–82.7)	0–100
MIDAS score, mean (SD, range)	38.6 (35.8, 0–240)	0–270
MIDAS, median (IQR)	27.0 (14.0–50.0)	0–270
DASS Depression score, mean (SD, range)	6.3 (4.7, 0–21)	0–21
DASS Anxiety score, mean (SD, range)	4.3 (3.4, 0–17)	0–21
DASS Stress score, mean (SD, range)	8.4 (4.2, 0–21)	0–21
PCS, mean (SD, range)	21.4 (11.5, 0–52)	0–52
PCS subscale rumination score, mean (SD, range)	8.1 (4.5, 0–16)	0–16
PCS subscale magnification score, mean (SD, range)	3.8 (2.6, 0–12)	0–12
PCS Subscale helplessness score, mean (SD, range)	9.6 (5.8, 0–24)	0–24
CHS adapted subscale fear of attacks score, mean (SD, range)	10.2 (3.0, 3–15)	3–15

Abbreviations: CHS, Cluster Headache Scales; DASS, Depression Anxiety and Stress Scales; IQR, interquartile range; MIDAS, Migraine Disability Assessment; PCS, Pain Catastrophizing Scale; PDI, Pain Disability Index (converted to percentage); SD, standard deviation.

### Zero‐order correlations between disability and headache and psychological outcome parameters

Zero‐order correlations between disability (MIDAS, PDI [converted to percentage]), headache characteristics (attack frequency, pain intensity), and psychological variables (pain catastrophizing, fear of attacks, depression, anxiety, stress) were calculated. The results are shown in Table 3.

**TABLE 3:** Zero‐order correlations between migraine‐related disability and psychological outcome parameters (*N* = 391). [Color table can be viewed at wileyonlinelibrary.com]

Outcome parameter	Attack frequency		Pain intensity		PDI score		MIDAS score		PCS score		Fear of attacks		DASS depression		DASS stress		DASS anxiety	
	*r*	*p*	*r*	*p*	*r*	*p*	*r*	*p*	*r*	*p*	*r*	*p*	*r*	*p*	*r*	*p*	*r*	*p*
Attack frequency	**1.00**	–	–	–	–	–	–	–	–	–	–	–	–	–	–	–	–	–
Pain intensity	0.06	0.254	**1.00**	–	–	–	–	–	–	–	–	–	–	–	–	–	–	–
PDI score	**0.29**	<0.001	**0.27**	<0.001	**1.00**	–	–	–	–	–	–	–	–	–	–	–	–	–
MIDAS score	**0.55**	<0.001	**0.19**	<0.001	**0.42**	<0.001	**1.00**	–	–	–	–	–	–	–	–	–	–	–
PCS score	**0.24**	<0.001	**0.24**	<0.001	**0.39**	<0.001	**0.32**	<0.001	**1.00**	–	–	–	–	–	–	–	–	–
Fear of attacks	**0.12**	0.023	**0.18**	<0.001	**0.31**	<0.001	**0.26**	<0.001	**0.31**	<0.001	**1.00**	–	–	–	–	–	–	–
DASS depression	**0.19**	<0.01	**0.11**	0.027	**0.44**	<0.001	**0.24**	<0.001	**0.60**	<0.001	**0.34**	<0.001	**1.00**	–	–	–	–	–
DASS stress	**0.16**	0.020	0.09	0.071	**0.42**	<0.001	**0.21**	<0.001	**0.46**	<0.001	**0.34**	<0.001	**0.71**	<0.001	**1.00**	–	–	–
DASS anxiety	**0.14**	0.060	**0.15**	0.030	**0.35**	<0.001	**0.23**	<0.001	**0.40**	<0.001	**0.37**	<0.001	**0.60**	<0.001	**0.58**	<0.001	**1.00**	–

*Note*: Significant correlations are marked in bold (two‐tailed); light blue, weak correlation; blue, moderate correlation; dark blue, strong correlation.

Abbreviations: Attack frequency, reported mean monthly migraine days; DASS, Depression Anxiety and Stress Scales; MIDAS, Migraine Disability Assessment; PCS, Pain Catastrophizing Scale; PDI, Pain Disability Index (converted to percentage); *r*, Pearson's rho, Spearman's rho used for correlations with attack frequency.

Pain intensity showed no significant association with attack frequency and the DASS stress subscale. Correlation was statistically significant for all other parameters. In terms of *disability*, the PDI (converted to percentage) showed a weak correlation with attack frequency, while the MIDAS score demonstrated a moderate correlation with this variable. Furthermore, the PDI (converted to percentage) exhibited moderate correlations with the DASS subscales (stress, anxiety, and depression) and fear of attacks, whereas these associations were weaker for the MIDAS. Regarding *pain catastrophizing*, moderate correlations were found with the PDI (converted to percentage) and MIDAS, and a strong correlation with fear of attacks.

### Path analyses for the prediction of migraine‐related disability

In Model 1, the relationships between attack frequency, pain intensity, pain catastrophizing, fear of attacks, depressiveness, and migraine‐related disability were examined, with disability assessed using the PDI (converted to percentage). The model demonstrated a good fit (*χ* ^*2*^ = 15.88, *df* = 7, *p* = 0.026, *χ* ^*2*^/*df = 2.27*, CFI = 0.98, RMSEA = 0.06, SRMR = 0.03). All path coefficients were significant (*p* < 0.01). The model accounted for 29% of the variance in migraine‐related disability (*R* ^*2*^ = 0.29, adjusted *R* ^*2*^ = 0.28), indicating a large effect. Depressiveness showed the strongest total effect on disability (0.34), followed by pain intensity (0.27), frequency of attacks (0.26), and fear of attacks (0.12).

Model 2, which used the MIDAS to assess disability, likewise showed a good fit (χ^2^ = 14.93, *df* = 7, *p* = 0.037, χ^2^/*df* = 2.13, CFI = 0.98, RMSEA = 0.05, SRMR = 0.03). Path analysis explained 30% of the variance in disability (*R* ^*2*^ = 0.30, adjusted *R* ^*2*^ = 0.28), indicating a large effect size. All path coefficients were significant (*p* < 0.05). In contrast to Model 1, frequency of attacks (0.48) showed the strongest total effect on disability, followed by pain intensity (0.20) and fear of attacks (0.13), with depressiveness (0.12) having the smallest effect.

In *both models*, pain catastrophizing significantly predicted fear of attacks (β = 0.60, *p* < 0.001) and depressiveness (β = 0.44, *p* < 0.001). The variance explained in both models for fear of attacks indicated large effect size (*R* ^*2*^ = 0.36), while depressiveness was predicted with medium effect size (*R* ^*2*^ = 0.19).

The *proportion of the total explained variance in disability attributable to mediation* was calculated as described in the methods sections, with results provided in the Appendix A. In path analysis 1, *R* ^*2*^_mediation_ = 0.073 and in path analysis 2, *R* ^*2*^_mediation_ = 0.064, both indicating small effect sizes. Relative to the total explained variance (*R* ^2^) of disability, this corresponds to mediation proportions of 25.2% (0.07 of 0.29) and 21.3% (0.06 of 0.30).

**FIGURE 3:**
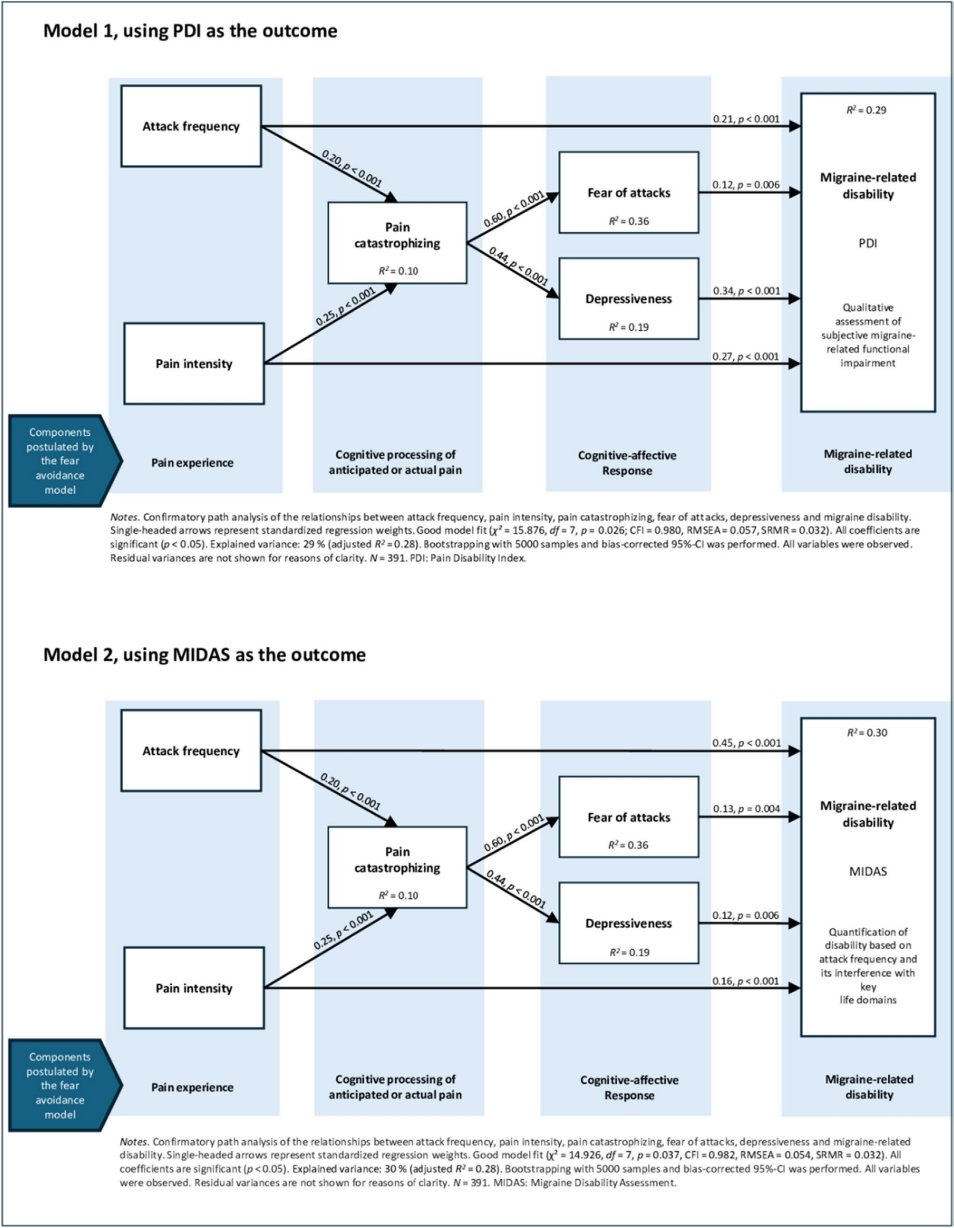
Contributing factors of disability in episodic migraine and their interactions, based on the theoretical framework of the fear‐avoidance model, with the Pain Disability Index (PDI, Model 1) or Migraine Disability Assessment (MIDAS, Model 2) used as the outcome. CFI, Comparative Fit Index; CI, confidence interval; *df*, degrees of freedom; RMSEA, root mean square error of approximation; SRMR, standardized root mean square residual. [Color figure can be viewed at wileyonlinelibrary.com]

The estimates and effects of both path analyses are summarized in Table 4. For a graphical representation of the path analyses, see Figure 3.

**TABLE 4:** Estimates and effects of the path analyses.

Model	Estimates unstandardized	95% CI^a^			Standardized effects		
		Lower bound	Upper bound	*p*	Total	Direct	Indirect
Model 1, dependent variable: PDI							
→ Disability							
Attack frequency	0.35	0.21	0.48	**<0.001**	0.26	0.21	0.05
Pain intensity	2.35	1.34	3.41	**<0.001**	0.27	0.22	0.06
Fear of attacks	0.70	0.14	1.27	**0.017**	0.12	0.12	–
Depressiveness	1.24	0.94	1.57	**<0.001**	0.34	0.34	–
Pain catastrophizing	–	–	–	–	0.22	–	0.22
→ Pain catastrophizing							
Attack frequency	0.22	0.12	0.33	**<0.001**	0.20	0.20	–
Pain intensity	1.80	1.10	2.51	**<0.001**	0.25	0.25	–
→ Fear of attacks							
Attack frequency	–	–	–	**–**	0.12	–	0.12
Pain intensity	–	–	–	**–**	0.15	–	0.15
Pain catastrophizing	0.16	0.14	0.18	**<0.001**	0.60	0.60	–
→ Depressiveness							
Attack frequency	–	–	–	**–**	0.09	–	0.09
Pain intensity	–	–	–	**–**	0.11	–	0.11
Pain catastrophizing	0.18	0.14	0.22	**<0.001**	0.44	0.44	–
Model 2, dependent variable: MIDAS							
→ Disability							
Attack frequency	1.54	1.25	1.85	**<0.001**	0.48	0.45	0.03
Pain intensity	3.70	1.96	5.60	**<0.001**	0.20	0.16	0.03
Fear of attacks	1.51	0.51	2.55	**0.004**	0.13	0.13	–
Depressiveness	0.91	0.10	1.71	**0.008**	0.12	0.12	–
Pain catastrophizing					0.13	–	0.13
→ Pain catastrophizing							
Attack frequency	0.22	0.12	0.33	**<0.001**	0.20	0.20	–
Pain intensity	1.80	1.10	2.51	**<0.001**	0.25	0.25	–
→ Fear of attacks							
Attack frequency		–	–	–	0.12	–	0.12
Pain intensity		–	–	–	0.15	–	0.15
Pain catastrophizing	0.16	0.14	0.18	**<0.001**	0.60	0.60	–
→ Depressiveness							
Attack frequency		–	–	–	0.09	–	0.09
Pain intensity		–	–	–	0.11	–	0.11
Pain catastrophizing	0.18	0.14	0.22	**<0.001**	0.44	0.44	–

*Note*: Assessment test, *N* = 391. Bold values statistically significant at *p* < 0.05.

Abbreviations: CI, confidence interval; MIDAS, Migraine Disability; PDI, Pain Disability Index (converted to percentage).

^a^ Bias‐corrected percentile method.

## DISCUSSION

This study was conducted to identify mechanisms underlying disability in episodic migraine. The FAM was applied as a theoretical framework for the investigation of interactions between cognitive‐affective factors. Path analyses demonstrated that both headache characteristics (attack frequency and pain intensity) and cognitive‐affective factors (fear of attacks and depressiveness) contribute to disability. As postulated by the FAM, pain catastrophizing emerged as an important mediator.

### Findings of this study in the context of the FAM

1. In addition to a direct link between pain experience and disability, an indirect pathway was found, which was mediated by pain catastrophizing. Pain catastrophizing was strongly associated with fear of attacks (explaining 36% of the variance) and depressiveness (explaining 19% of the variance). The effects of these relationships were among the strongest in both path analyses.
2. Fear of attacks and depressiveness were found as independent predictors of disability, in addition to attack frequency and pain intensity. Fear of attacks was common in the study population: More than half (57.8%) reported worry and about a third (30.7%) reported panic when they thought of a future attack.
3. The influence of psychological factors on disability was significant. The mediation via pain catastrophizing accounted for 20–25% of the total explained variance, depending on the measurement instrument used. The absolute mediation effect was small, but comparable to the contribution of attack frequency observed in the MIDAS evaluation studies.^9^

Taken together, these results demonstrate the substantial impact of psychological factors on disability and highlight its multifactorial nature, even in episodic migraine. The interaction between pain experience, pain processing, cognitive‐affective responses, and disability are in line with the theoretical assumptions of the FAM. A strong relationship between pain catastrophizing, fear of pain, depression, and disability has already been demonstrated in various pain disorders, as well as primary headache disorders.^48^, ^49^ This suggests that these transdiagnostic factors also reflect important mechanisms for disability in migraine.

A recent meta‐analysis^48^ that examined the cross‐sectional association between the primary components of the FAM in clinical pain studies found positive, medium to large associations between pain catastrophizing, fear of pain, pain vigilance, and anxiety, as well as depression and pain‐related disability. Differences in effect sizes suggest that pain catastrophizing, which is a key factor in the postulated cascade of the FAM, is more strongly associated with all outcomes than fear of pain, or pain hypervigilance.

Originally developed for chronic lower back pain, the FAM has been used to develop therapeutic approaches for various pain conditions (e.g., as described by Zale and Ditre^50^). In one of our previous studies,^49^ the FAM was applied to cluster headache. We found that fear of attacks contributed more to disability than attack frequency and remained an independent predictor even when controlling for depression and anxiety. Regarding migraine, research has primarily focused on modifying dysfunctional trigger‐avoidance behavior driven by fear of attacks.^18^, ^51^, ^52^ The integration of this approach into a cognitive behavioral therapy program showed potential, but effectiveness has yet to be conclusively demonstrated.^53^

### Pain catastrophizing: Well‐known, but still not fully understood

This study identified pain catastrophizing as a critical mediator of disability, consistent with existing research. Studies in migraine have showed that pain catastrophizing is linked to more frequent and severe headache, increased psychological distress, and disability.^54^, ^55^, ^56^, ^57^, ^58^ Studies on chronic pain treatments also identified pain catastrophizing as a critical factor in therapeutic success.^59^ Psychotherapeutic approaches, such as mindfulness, acceptance, and cognitive restructuring, effectively reduce both disability and pain catastrophizing.^60^, ^61^, ^62^, ^63^, ^64^, ^65^, ^66^, ^67^, ^68^, ^69^ While the mechanisms remain unclear, these findings underscore pain catastrophizing as a key therapeutic target for alleviating disability in migraine.^70^

Pain catastrophizing, which has been empirically demonstrated to be modifiable,^59^, ^71^ is a dysfunctional cognition frequently present in patients with migraine;^58^ however, there is a notable lack of studies examining its causal effects. To our knowledge, only one study^60^ in migraine evaluated the efficacy of a cognitive behavioral therapy specifically designed to reduce pain catastrophizing. That randomized controlled trial (RCT) demonstrated that cognitive restructuring effectively reduced pain catastrophizing and led to significant improvements in anxiety, depression, and migraine‐related disability, as well as self‐efficacy. However, only patients with chronic migraine were included in that study. Further RCTs in episodic and chronic migraine are needed to better understand the impact of pain catastrophizing. These studies should investigate its ictal and interictal effects on disability, as well as its interaction with other psychological factors and headache characteristics.

### Implications for the assessment of disability

This study was conceptualized to assess disability as a multifaceted construct encompassing both subjective and quantitative dimensions. The findings of this study revealed notable differences depending on the assessment tool employed. With the MIDAS, which quantifies disability based on attack frequency and its interference with key life domains, attack frequency emerged as the strongest predictor. In contrast, the PDI (converted to percentage), reflecting a more qualitative and subjective patient perspective, identified depressiveness as the strongest predictor. These discrepancies echo findings by Klonowski et al.,^72^ who also reported that psychological variables were more strongly linked to the subjective degree of headache‐related disability (PDI, converted to percentage) than quantitative measures (MIDAS). This underlines the importance of selecting or combining instruments based on specific research or clinical focus.

### Limitations

Certain limitations need to be considered while examining the results of this study. As the study was cross‐sectional, it precludes establishing causal relationships between the investigated variables. It is also important to note that mediation analyses in cross‐sectional data can yield biased estimates of direct and indirect effects, as they exclude autoregressive paths, inflating cross‐lagged estimates. Moreover, the assessment of fear of attacks was undertaken using an adaptation of the corresponding subscale from the CHS questionnaire, originally designed for cluster headache. While its generic item formulation appeared suitable for migraine, future research should use migraine‐specific tools to improve measurement precision, like the Fear of Attacks in Migraine Inventory.^17^ Regarding the sample, reported female sex was markedly overrepresented, accounting for 94% of the sample. Female sex is associated with higher attack frequency, longer attack duration, a prolonged recovery period, and greater disability, as well as higher prevalence of psychiatric comorbidities,^15^, ^73^ which limits the generalizability of the results. Furthermore, most participants reported a MIDAS level of III or IV, which corresponds to moderate to severe disability. Identifying mechanisms of disability is particularly important in this population, as migraine‐specific psychological interventions are generally not required for patients with low disability. Nevertheless, the study design has some strengths. Mediation analyses can help to improve the understanding of interactions between different factors but have received little attention in migraine research.^74^ Moreover, the theory‐driven approach integrates biological, cognitive, and affective factors evident in migraine, and provides a solid framework that facilitates the development of an explanatory model.

## CONCLUSION

The results demonstrate the substantial impact of psychological factors on disability in migraine and underline its multifactorial nature. The results of this study provide insights into the interactions between cognitive‐affective factors and headache characteristics, and provide empirical evidence for the theoretical assumptions of the FAM. In addition to the pain experience, dysfunctional cognitive pain processing appears to be an important mediator in the genesis of disability. Moreover, in the comprehensive assessment of disability within a research or clinical context, assessment instruments should be carefully selected or combined, as they capture different aspects of disability. Further RCTs designed to examine the causal effects of pain catastrophizing have the potential to improve the understanding of interactions and mechanisms.

## AUTHOR CONTRIBUTIONS

**Janosch Fox:** Conceptualization; data curation; formal analysis; funding acquisition; investigation; methodology; project administration; visualization; writing – original draft; writing – review and editing. **Charly Gaul:** Supervision; writing – review and editing. **Merle Kuhlencord:** Writing – review and editing. **Nicolina Peperkorn:** Writing – review and editing. **Julia Ohse:** Writing – review and editing. **Joshua Krutzki:** Writing – review and editing. **Youssef Shiban:** Supervision; validation; writing – review and editing.

## FUNDING INFORMATION

We acknowledge support from the Open Access Publication Fund of the Medical Faculty of the University of Duisburg‐Essen.

## CONFLICT OF INTEREST STATEMENT

**Janosch Fox, Merle Kuhlencord, Nicolina Peperkorn, Julia Ohse, Joshua Krutzki**, and **Youssef Shiban** declare no conflict of interest. **Charly Gaul** has received honoraria for consulting and lectures within the past 3 years from Abbvie, Lilly, Novartis Pharma, Hormosan Pharma, Sanofi‐Aventis, Lundbeck, Perfood, Vectura Fertin Pharma, Chordate, Pfizer, Dr. Reddys, Merz, Reckitt‐Benckiser, and TEVA. His research is supported by a grant from the German Research Foundation (DFG). He does not hold any stocks in pharmaceutical companies.

## APPENDIX A

Results of the multiple linear regression analyses for the variance analysis of pain‐related disability, assessed with the Pain Disability Index (converted to percentage) as outcome, *N* = 391.

**Table:**

Model	*R*	*R* ^*2*^	*R* ^*2*^_*adj*_	*p*	SE_*B*_	*B*	β
Model 1	0.48	0.23	0.23	<0.001			
Intercept				<0.001	3.54	14.27	
Attack frequency				<0.001	0.08	0.37	0.22
Pain intensity				<0.001	0.50	2.22	0.20
Pain catastrophizing				<0.001	0.07	0.45	0.30
Model 2	0.39	0.15	0.15	<0.001			
Intercept				<0.001	1.70	32.23	
Pain catastrophizing				<0.001	0.07	0.59	0.39
Model 3	0.30	0.15	0.145	<0.001			
Intercept				<0.001	3.70	16.90	
Attack frequency				<0.001	0.08	0.47	0.29
Pain intensity				<0.001	0.51	3.04	0.28

Abbreviations: adj, adjusted; SE, standard error.

Results of the multiple linear regression analyses for the variance analysis of pain‐related disability, assessed with the Migraine Disability Assessment as outcome, *N* = 391.

**Table:**

Model	*R*	*R* ^*2*^	*R* ^*2*^_*adj*_	*p*	SE_*B*_	*B*	β
Model 1	0.53	0.28	0.28	<0.001			
Intercept				<0.001	7.81	−28.72	
Attack frequency				<0.001	0.17	1.58	0.42
Pain intensity				<0.001	1.11	3.92	0.16
Pain catastrophizing				<0.001	0.16	0.67	0.20
Model 2	0.32	0.10	0.10	<0.001			
Intercept				<0.001	3.99	16.22	
Pain catastrophizing				<0.001	0.16	1.07	0.32
Model 3	0.50	0.25	0.24	<0.001			
Intercept				0.002	7.93	−24.81	
Attack frequency				<0.001	0.17	1.73	0.46
Pain intensity				<0.001	1.10	5.13	0.21

Abbreviations: adj, adjusted; SE, standard error.
